# GCN2 upregulates autophagy in response to short-term deprivation of a single essential amino acid

**DOI:** 10.1080/27694127.2022.2049045

**Published:** 2022-04-07

**Authors:** Anne-Catherine Maurin, Laurent Parry, Wafa B’chir, Valérie Carraro, Cécile Coudy-Gandilhon, Ghita Chaouki, Cédric Chaveroux, Sylvie Mordier, Brigitte Martinie, Vanessa Reinhardt, Céline Jousse, Alain Bruhat, Patrice Codogno, Julien Averous, Pierre Fafournoux

**Affiliations:** a Unité de Nutrition Humaine, INRAE, Université Clermont Auvergne, UMR 1019, F-63000 Clermont-Ferrand, France; b Centre de Recherche en Cancérologie de Lyon, INSERM U1052, CNRS 5286, Centre Léon Bérard, Université Lyon, F-69373, Lyon, France; c Plateau Technique de Microscopie Electronique, INRAE, F-63122 Saint-Genès-Champanelle, France; d Université Paris Descartes-Sorbonne Paris Cité, Paris F-75006, France; e Institut Necker-Enfants Malades, INSERM U1151-CNRS UMR 8253, Paris F-75993, France

**Keywords:** ATF4, eIF2α, leucine, lysine

## Abstract

The ability to adapt the proteolysis rates based on fluctuations in essential amino acid (EAA) availability is essential for life. The GCN2-eIF2α-ATF4 signaling pathway is involved in the adaptive response to EAA deprivations. Our previous results demonstrated that activation of this pathway is involved in upregulating the expression of many autophagy genes at the transcriptional level thanks to the transcription factor ATF4. In the present study, we investigated whether the kinase GCN2 is also involved in early upregulation of the autophagic process in response to EAA deficiencies, thereby contributing to a rapid increase in the proteolysis rate. We observed that a one-hour leucine deprivation upregulated autophagy in both cultured cells and *in vivo* in mouse liver, as reflected by an increase in both [S278]-ATG16L1 phosphorylation and LC3B conversion, and decreased p62 protein level. Using cells and mice with genetic ablation of *Gcn2* as well as genetic reconstitution experiments *in vitro*, data showed that GCN2 was required for this upregulation of autophagy. Downstream GCN2, the phosphorylation of eIF2α was necessary, while ATF4 was not. Overall, these findings revealed a major role of GCN2-eIF2α signaling in the regulation of autophagy in response to short-term EAA deprivation.

## Introduction

In healthy adults, nine “essential amino acids” (EAA) cannot be synthesized by the organism, and must be supplied by food. To cope with fluctuations in dietary proteins/AA availability, mammals have evolved a broad spectrum of adaptive mechanisms. They first sense and then respond to variations in EAA availability. For example, the consumption of a diet deficient in a single EAA produces a rapid and significant decrease in circulating levels of the missing EAA [1–3]. The drop in blood AA concentration is then sensed by the organism which responds by an innate aversion to EAA-imbalanced food [2,^4–6^]. This type of nutritional situation is frequently observed in omnivorous animals living in the wild. Furthermore, alongside variations in the availability of dietary nutrients, the body may experience significant changes in AA profiles as a result of pathological conditions. A number of diseases are associated with a catabolic state and deregulated AA homeostasis [7,8]. Since proteins must be permanently renewed throughout the body, EAA deficiencies are recognized as a stress, and require the organism to adapt rapidly.

In response to AA deficiencies, one of the adaptive responses of the organism consists in hydrolyzing its own proteins to produce free AA [9–12]. Pioneering studies demonstrated that the first tissue to hydrolyze resident proteins is the liver, through upregulation of macro-autophagy (hereafter referred to as autophagy) [10,12,^13^]. This degradation process involves the engulfment of cytoplasmic components within double-membrane vesicles that ultimately fuse with lysosomes [10,^14–16^]. Autophagy involves about 38 autophagy-related genes (*Atgs*) which generate multiprotein complexes acting sequentially [17–19]. In addition to basal functioning to ensure the constitutive turnover of cellular components, this process may be mobilized and upregulated in response to numerous stressors [20–22].

The autophagic process is regulated by nutrient availability, including access to AAs, notably through modulation of the kinase activity of mTORC1 (serine/threonine-protein kinase mTOR complex 1) [23–26]. Indeed, mTORC1 is active and restrains autophagy in nutrient-rich conditions, whereas its inhibition upon nutrient deprivation derepresses autophagy. mTORC1 downregulates autophagy in particular by phosphorylating serine 757 in ULK1 (serine/threonine-protein kinase ULK1) [27,28], which is part of one of the most upstream complex (ULK complex) involved in initiating formation of the double-membrane phagophore [29–34]. Alongside mTORC1, the protein kinase GCN2 (eIF-2-alpha kinase GCN2) also senses intracellular changes in AA availability [35–37]. Whereas mTORC1 is activated in response to AA supply to promote anabolism and to inhibit catabolism, GCN2 is specifically activated by EAA scarcities, and promotes adaptation of the cells to nutritional stress conditions. In response to EAA deficiency, GCN2 links the accumulation of uncharged tRNAs to eIF2α (eukaryotic translation initiation factor 2 subunit alpha) phosphorylation on serine 51 [38]. This activity allows GCN2 to reduce the overall protein synthesis rate, while simultaneously activating a gene expression program mediated by the translationally upregulated transcription factor ATF4 (cyclic AMP-dependent transcription factor ATF-4) [37,^39^,^40^]. In a previous study, Talloczy et al. demonstrated that eIF2α signaling regulates autophagy in fibroblasts in response to either withdrawal of all AAs or viral infection [41]. More recently, the expression of a number of *Atgs* was found to be transcriptionally regulated by ATF4 [42,43], an effect that can be assumed to contribute to maintaining a high level of autophagy during EAA deprivations. However, most cells express relatively high levels of ATG proteins under physiological conditions, ensuring a basal level of autophagy and allowing the process to be upregulated within minutes in cultured cells [44–46]. Given that the timeline for activation of the stress kinase GCN2 is also measured in minutes in response to EAA deficiencies [47,48], we wondered whether GCN2 could also play a role in the early events of autophagy upregulation in response to deprivations of a single EAA.

In the study presented here, we investigated the role of GCN2 in regulating autophagy in response to short-term deprivations of a single EAA. Our data show that leucine deprivation induces an increased recruitment of newly-forming autophagosomes and the whole process of autophagy in a GCN2-dependent manner, both in cultured cells and mouse liver. This effect does not require ATF4 expression but involves the phosphorylation of eIF2α. Furthermore, in response to short-term leucine starvation, the induction of autophagy is associated with a GCN2-dependent decrease in mTORC1 activity, unlike short-term lysine deprivation that requires GCN2 for inducing autophagy, without an associated decrease in either [T389]-S6K or [S757]-ULK1 phosphorylation by mTORC1. Overall, our results indicate that GCN2 plays a major role in promoting early events of autophagy upregulation in response to EAA shortages.

## Results

### GCN2 is required for upregulating autophagy in response to short-term leucine deficiency

**Figure 1.**
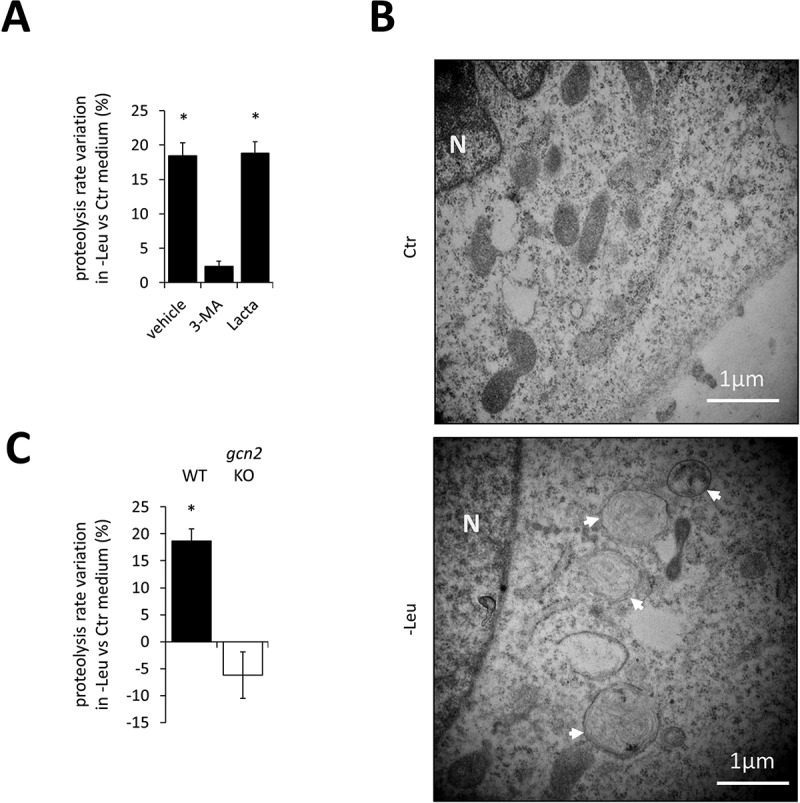
Leucine deprivation rapidly triggers an autophagy-dependent upregulation of protein breakdown that requires GCN2. (**A**) The upregulation of proteolysis during leucine deprivation results from mobilization of the autophagic process. Protein degradation was measured using L-[35S]Met radiolabeling of cellular proteins and pulse-chase approach. Radio-labeled L-[^35^S]Met wild-type (WT) MEFs were incubated in the control (Ctr) or leucine-devoid (-Leu) medium for 2 h without inhibitor (vehicle) or in the presence of either an inhibitor of autophagy (3-methyladenine; *3-MA*, 10 mM), or an inhibitor of proteasome-dependent proteolysis (lactacystin; *Lacta*, 2 µM). Three independent experiments were performed. Results are expressed as variation in proteolysis rate in response to leucine deprivation compared to control proteolysis rate (control medium). Bar values are mean ± SEM (*, p < 0.05 relative to the control, Student’s t-test). (**B**) Electron microscopy images of autophagic structures (arrows) following incubation of WT MEFs in the Ctr or -Leu medium for 2 h. (**C**) Impact of *Gcn2* knock-out (KO) on variations in proteolysis resulting from leucine deprivation. WT and *gcn2* KO MEFs were incubated in the Ctr or -Leu medium for 2 h, and protein degradation was measured as described for (A). Three independent experiments were performed and results were analyzed and expressed as in (A).

To characterize the role of GCN2 in the proteolytic response to short-term deprivations of a single EAA, we used a leucine-starved mouse embryonic fibroblasts (MEFs) culture model. We first analyzed how leucine deprivation affected protein breakdown using L-[^35^S]Met radio-labeling experiments, as previously described [49]. The lack of leucine in the culture medium induced a higher release of L-[^35^S]Met as compared with control medium. A 2-h leucine deprivation increased the proteolysis rate by 18% in wild-type (WT) MEFs (Fig. 1A). This effect was almost completely abolished in the presence of 3-methyladenine (an inhibitor of autophagy [50]) whereas lactacystin (an inhibitor of the proteasome [51]) had no effect (Fig. 1A). These results suggest that the increase in proteolysis was mainly due to autophagy. In support of this hypothesis, ultrastructural analysis revealed the presence of a large number of autophagic structures in the cytoplasm of leucine-deprived WT MEFs (Fig. 1B). Subsequently, to determine the role of GCN2 in this process, we used *gcn2* knock-out (KO) MEFs. No increase in proteolysis in response to leucine deficiency was observed in *gcn2* KO MEFs (Fig. 1C), demonstrating that GCN2 was required to trigger this process.

To analyze the role of GCN2 in the upregulation of autophagy, we took advantage of several protein markers for monitoring autophagy. We first analyzed the recently described specific marker of autophagy induction, ATG16L1 (autophagy-related protein 16- 1) phosphorylation on Ser278 [52]. Tian et al. reported that phospho-[S278]-ATG16L1 is only present on newly-formed autophagosomes, thus [S278]-ATG16L1 phosphorylation monitoring can be efficiently used to measure autophagy induction [52,^53^]. We also analyzed LC3B (microtubule-associated protein 1 light chain 3 beta) protein lipidation (hereafter called LC3), a well-recognized marker of autophagosome formation [53,^54^] (conversion of the cytosolic form LC3-I to the lipid-bounded and autophagosome-anchored LC3-II). Finally, we measured the level of p62 protein (also called SQSTM1 for sequestosome-1), a well-known autophagy substrate the decrease of which can be interpreted as an increase in the autophagy-dependent degradation rate [53].

**Figure 2.**
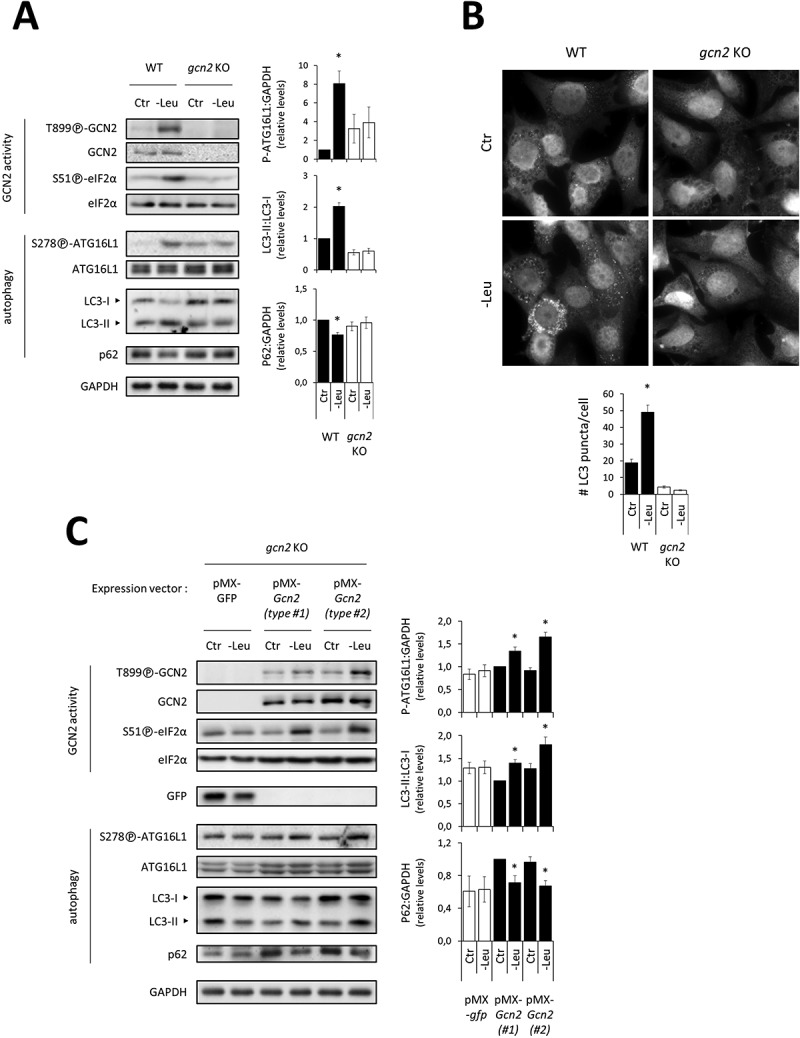
GCN2 is required for upregulating autophagy in response to short-term leucine deprivation in MEFs. (**A**) Immunoblot analyses of total protein extracts of WT and *gcn2* KO MEFs either incubated in the Ctr or -Leu medium for 1 h. Representative immunoblots and quantification of relative intensities of P-[S278]-ATG16L1 to GAPDH, LC3-II to LC3-I and p62 to GAPDH are shown (four independent experiments). Bar values are mean ± SEM (*, p < 0.05 relative to Ctr of the same cell type, Student’s t-test). As previously observed by others [52], the anti-ATG16L1 antibody detected two isoforms (**B**) Representative images and quantification of LC3-labeled punctae. WT and *gcn2* KO MEFs were cultured in the Ctr or -Leu medium in the presence of 20 µM chloroquine for 2 h. Endogenous LC3 was detected by immunofluorescence and average number of punctae per cell was determined (40-50 cells were analyzed per condition in three independent experiments). Bar values are mean ± SEM (*, p < 0.05 relative to Ctr of the same cell type, Student’s t-test). (**C**) Immunoblot analyses of total protein extracts of *gcn2* KO MEFs overexpressing either GFP as a control or GCN2. Two pMX-*Gcn2*-transduced populations are shown, one total population (type #1) and one clone (type #2). Cells were either incubated in the Ctr or -Leu medium for 2 h. Representative immunoblots and relative quantifications of LC3-II to LC3-I and P-[S278]-ATG16L1 and p62 to GAPDH are given (three independent experiments). Bar values are mean ± SEM (*, p < 0.05 relative to Ctr of the same cell type, Student’s t-test).

In WT MEFs, we observed that in addition to activating GCN2 (as indicated by self-phosphorylation of GCN2 and phosphorylation of eIF2α), leucine withdrawal upregulated the phosphorylation of [S278]-ATG16L1 and the conversion of LC3B, while causing a decrease in p62 protein level, as early as 1 h after the onset of deprivation (Fig. 2A). Thus, short-term leucine deprivation resulted in an increased recruitment of newly formed autophagosomes and an increased rate of autophagic degradation. Marker analysis also indicated upregulation of autophagy at 2 h and 4 h of leucine deprivation (Fig. S1). None of these effects were observed in *gcn2* KO cells (Fig. 2A and S1). Furthermore, results from LC3 conversion analysis in the presence of chloroquine, an inhibitor of autophagosome-lysosome fusion, confirmed that the autophagic flux was increased in response to 1 h leucine deprivation, an effect that was compromised in *gcn2* KO cells (Fig. S2). The inability of *gcn2* KO MEFs to activate LC3 conversion in response to leucine deprivation was confirmed by LC3 immunofluorescence (Fig. 2B). In response to leucine deficiency, the number of LC3 punctae corresponding to membrane-anchored LC3-II localizing to autophagic structures was increased in WT cells. No modification of LC3 labeling was observed in *gcn2* KO cells (Fig. 2B). All these data are consistent with those obtained from protein breackdown measurements (Fig. 1). Thus, *Gcn2* ablation prevented upregulation of the autophagic process resulting from leucine deficiency in MEFs. In order to test whether the reacquisition of GCN2 function in *gcn2* KO MEFs could be sufficient to confer them the ability to upregulate autophagy in response to leucine deprivation, we performed a genetic reconstitution experiment by stably expressing GCN2 (or GFP as a negative control) in *gcn2* KO cells. Fig. 2C shows that in response to leucine deficiency, [S278]-ATG16L1 phosphorylation and LC3 lipidation were increased whereas p62 level was decreased in KO cells re-expressing GCN2 but not in KO cells expressing GFP.

Taken together, these results show a major role of GCN2 in the induction of autophagy resulting from leucine deprivation in cultured cells.

**Figure 3.**
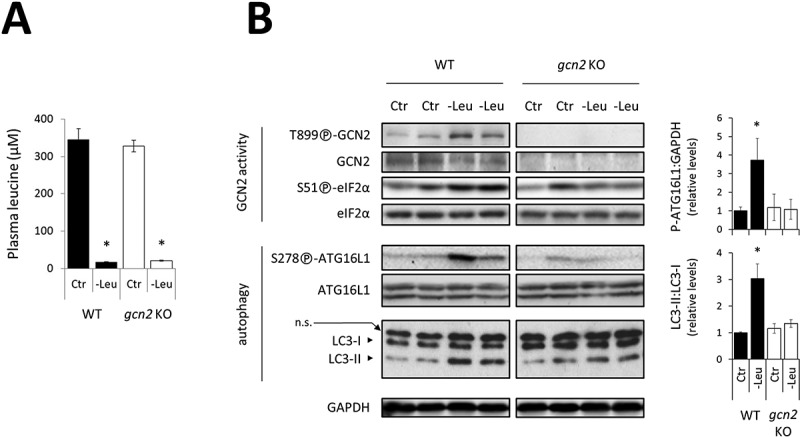
GCN2 activity is required to upregulate autophagy in response to leucine deficiency in mouse liver. After overnight fasting, WT and *gcn2* KO mice were fed the Ctr or -Leu experimental diet for 1 h. (**A**) Plasma leucine concentrations. Bar values are mean ± SEM (*, p < 0.05 relative to the Ctr for the same genotype, Student’s t-test; n=3). (**B**) Representative immunoblots of liver total protein extracts and relative quantifications of LC3-II to LC3-I and P-[S278]-ATG16L1 to GAPDH are given (6 mice per group from two independent experiments). Bar values are mean ± SEM (*, p < 0.05 relative to the Ctr for the same genotype, Student’s t-test). It is noticeable that the batch of anti-LC3B antibody used here detected a non-specific protein (n.s.) above LC3-I signal.

We then tested whether GCN2 was also required to upregulate autophagy *in vivo* in response to a dietary deficiency of a single EAA. We used the model of mice fed an AA-imbalanced diet following an overnight fast to induce a rapid drop in the blood concentration of the limiting EAA [2,3,^55^]. WT and *gcn2* KO mice were fed either a control or a leucine-free diet for 1 h. The mice fed the leucine-free diet showed a marked decrease in blood leucine content, regardless of their genotype (Fig. 3A). In the liver of WT mice, this effect was associated with GCN2 activation and marked upregulation of [S278]-ATG16L1 phosphorylation and LC3 conversion, indicating a significant induction of hepatic autophagy (Fig. 3B). In contrast, in livers from *gcn2* KO mice, the expression level of autophagy markers was not significantly affected by the leucine-free diet. Thus, our data indicate that GCN2 is required to upregulate hepatic autophagy in response to a dietary leucine deficiency.

Taken together, these results clearly establish that GCN2 is required to induce autophagy in response to leucine deprivation.

### eIF2α phosphorylation is required for the GCN2-dependent induction of autophagy, whereas ATF4 is dispensable

**Figure 4.**
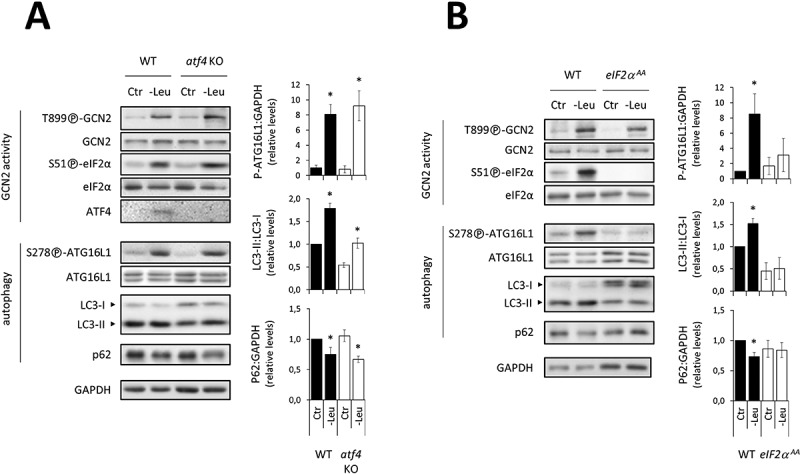
In response to short-term leucine deficiency, eIF2α phosphorylation is an essential event, whereas ATF4 is not required to upregulate autophagy. Immunoblot analyses of total protein extracts of WT and *atf4* KO MEFs (**A**) and eIF2α ^S51A/S51A^ (eIF2α ^AA^) MEFs (**B**) either incubated in the Ctr or -Leu medium for 1 h. In each case, representative immunoblots and relative quantifications of LC3-II to LC3-I and P-[S278]-ATG16L1 and p62 to GAPDH are shown (four independent experiments). Bar values are mean ± SEM (*, p < 0.05 relative to Ctr of the same cell type, Student’s t-test).

Following GCN2 activation, eIF2α phosphorylation stimulates the selective translation of ATF4, which activates a stress-related gene expression program. Our previous results demonstrated the essential role played by ATF4 in upregulating autophagy-related-gene transcription in response to a leucine deficiency in cultured cells [43]. However, for most of the genes studied, this was only detectable at least 2 h after leucine deprivation was initiated, suggesting that an increase in autophagy gene expression is not required to upregulate the autophagic process during the first 2 h following GCN2 activation. To test this hypothesis, we first investigated whether short-term leucine deprivation upregulated autophagy in *atf4* KO MEFs. As expected, a 1 h leucine deprivation led to phosphorylation of GCN2 and eIF2α in both WT and *atf4* KO MEFs (Fig. 4A). Furthermore, [S278]-ATG16L1 phosphorylation and LC3 lipidation were increased whereas p62 level was decreased in both cell lines (Fig. 4A). Thus, leucine deprivation increased autophagy in *Atf4* KO cells. Using a knock-down approach with an *Atf4*-specific siRNA, we confirmed that ATF4 was not required for upregulating autophagy in response to a lack of one EAA (Fig. S3).

We next examined whether eIF2α phosphorylation was necessary to the upregulation of autophagy associated with GCN2 activation. To do so, we tested the ability of MEFs expressing a non-phosphorylatable eIF2α protein (bearing mutation S51A) to activate autophagy in response to leucine deprivation for 1 h. In these cells (eIF2α ^AA^), the increase in [S278]-ATG16L1 phosphorylation and LC3 conversion and the decrease in p62 level associated with GCN2 activation were blunted (Fig. 4B).

Overall, our data indicate that eIF2α phosphorylation is necessary to the GCN2-dependent early upregulation of autophagy whereas ATF4 is not required.

### Short-term GCN2-dependent upregulation of autophagy is not systematically associated with decreased phosphorylation of [S757]-ULK1 and [T389]-S6K1

**Figure 5.**
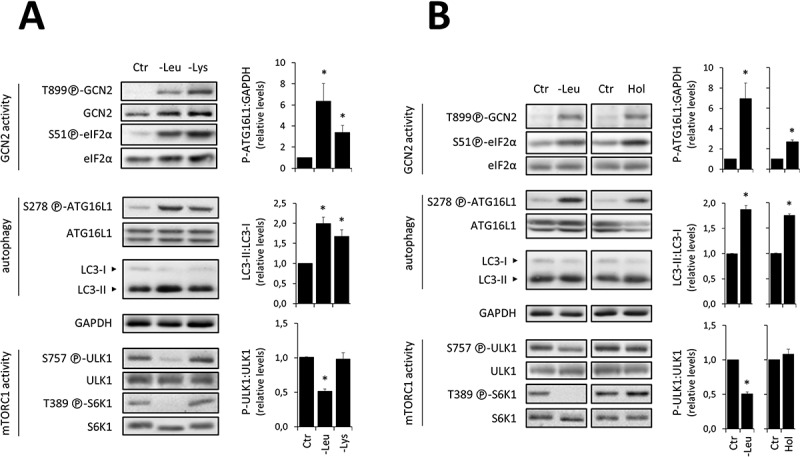
The upregulation of autophagy associated to GCN2 activation can occur without decreasing the phosphorylation level of either [S757]-ULK1 or [T389]-S6K1. Immunoblot analyses of total protein extracts from WT MEFs exposed for 1 h to the Ctr, -Leu or lysine-devoid (-Lys) medium (**A**) or L-histidinol-containing- (Hol, 4 mM) medium (**B**). In each case, representative immunoblots and relative quantifications of P-[S278]-ATG16L1 to GAPDH, LC3-II to LC3-I and P-[S757]-ULK1 to ULK1 are given (three independent experiments). Bar values are mean ± SEM (*, p < 0.05 relative to Ctr, Student’s t-test).

Our data clearly show that GCN2 is required for inducing autophagy in response to leucine deprivation. However, short-term leucine starvation was previously shown to inhibit mTORC1, the other AA sensing kinase that controls autophagy, and this effect was found to involve GCN2 [47,48,^56^,^57^]. We therefore investigated whether the GCN2-dependent induction of autophagy in response to leucine deprivation was associated with mTORC1 inhibition. As shown in Fig. 5 and S4, 1 h leucine deprivation led to both GCN2 activation and mTORC1 inhibition (as reflected by decreased levels of phospho-[T389]-S6K1 and phospho-[S757]-ULK1) in WT MEFs, and was associated with increased [S278]-ATG16L1 phosphorylation and LC3 conversion. Consistently with our previous data [47], the decrease in mTORC1 activity resulting from a 1 h leucine deficiency did not occur in *gcn2* KO cells (Fig. S4). This result indicates that in response to leucine deprivation, GCN2 could promote autophagy induction at least partly by inhibiting mTORC1.

To determine whether the GCN2-dependent induction of autophagy was or not always associated with mTORC1 inhibition, we sought GCN2 activators other than leucine deprivation. Published data indicate that different individual EAAs exhibit distinct effects on mTORC1 kinase activity [58–61]. As short-term lysine deficiency has been shown not to affect mTORC1 activity in MEFs [47], we deprived MEFs of lysine for 1 h (Fig. 5A). In these conditions, GCN2 was activated, and an increase in both [S278]-ATG16L1 phosphorylation and LC3 conversion was observed. However, neither the phospho-[T389]-S6K1:S6K1 nor the phospho-[S757]-ULK1:ULK1 ratios were decreased upon 1h exposure to lysine-deficient medium (Fig. 5). These results suggest that the upregulation of autophagy associated with GCN2 activation can occur without a concomitant decrease in the phosphorylation rate of [T389]-S6K1 and [S757]-ULK1.

To corroborate these results, we set up an additional experimental model in which we pharmacologically activated GCN2 with L-histidinol. This structural analogue of histidine inhibits loading of the histidyl-tRNA [62], thereby resulting in an increased concentration of free histidyl-tRNA without any associated AA deficiency. A 1-h treatment of MEFs with L-histidinol activated GCN2 and increased [S278]-ATG16L1 phosphorylation and LC3 lipidation, but did not elicit measurable decrease in phospho-[T389]-S6K1:S6K1 and phospho-[S757]-ULK1:ULK1 ratios (Fig. 5B). We even noticed an increase in [T389]-S6K1 phosphorylation following L-histidinol treatment, in accordance with previous studies [47,^63^]. Thus, our data show that short-term GCN2 activation can promote the induction of autophagy without decreasing phosphorylation of either [T389]-S6K1 or [S757]-ULK1 by mTORC1.

Overall, our results indicate that in addition to increasing the transcription level of numerous *Atg* via ATF4, the activation of GCN2 plays a major role in rapidly upregulating the autophagic process, including the induction step, in response to deprivations of a single EAA. This mechanism requires eIF2α phosphorylation but does not involve ATF4, and may or may not be related to a decrease in ULK1-Ser757 phosphorylation by mTORC1, depending on the nature of the EAA missing from the nutrient source.

## Study limitations

Although our results clearly show that GCN2 plays a key role in autophagy regulation in response to short-term EAA deprivation, we have not identified the precise molecular mechanism(s) involved. Identifying these mechanisms are beyond the scope of this paper and will be the subject of further studies. In the meantime, we believe that our work supports an important idea that GCN2 plays an essential role in promoting autophagy regulation in response to deprivation of a single EAA.

## Discussion

The ability to detect and respond to fluctuations in nutrient availability is essential for life. This is especially important in the case of consumption of a food totally or partially devoid of one EAA (EAA-imbalanced diet), which may result in conflicting effects on protein homeostasis. On the one hand, consumption of such a diet causes an increase in the content of all amino acids in the blood (except for the missing EAA), and would normally tends to favor anabolic processes. On the other hand, the EAA-imbalanced meal depletes circulating levels of the missing EAA, then activates adaptive mechanisms normally favoring catabolic processes. Finally, the consumption of EAA-imbalanced diets will predominantly inhibit protein synthesis and activate proteolysis. At the molecular level, these processes are primarily controlled by the AA-sensitive kinases GCN2 and mTORC1 [64]. In recent years, these two pathways were clearly demonstrated to regulate autophagy. mTORC1 exerts an accelerator/brake role via the post-translational control of activities of ULK complex and transcription factor EB [27,28,^65–68^] whereas GCN2 promotes the upregulation of numerous *Atgs* expression at the transcriptional level through its control of ATF4 [43]. Our current data demonstrate that GCN2 also promotes non-transcriptional steps of autophagy upregulation *in vitro* and *in vivo* in the liver.

The results presented here show that the GCN2-dependent upregulation of autophagy during short-term deprivations of one EAA requires the phosphorylation of eIF2α but not the expression of ATF4, suggesting possible involvement of translational regulations. A mechanism that could be considered is that a rapid overexpression of regulatory proteins by translational derepression of upstream open reading frame (uORF)-bearing mRNAs could be required (often promoted by eIF2α phosphorylation). The identification of posttranscriptional events triggered by GCN2-eIF2α signaling and promoting autophagy induction will require further researches.

Recently, Tian et al. have clearly demonstrated that ATG16L1 phosphorylation on Ser278 is a robust marker of autophagy induction [52]. Our results show that phosphorylation of this residue is increased during leucine deficiency. Interestingly, [S278]-ATG16L1 has been characterized as a target of ULK1 [69], which is part of the ULK complex, an important regulator of autophagosome formation [19,34]. This observation is consistent with previous published results by Jung et al. evidencing a high kinase activity of ULK1 in condition of leucine starvation [70]. As it was shown that short-term leucine deprivation was associated with a GCN2-dependent inhibition of mTORC1 activity ([47]; Fig. S4), the inhibition of mTORC1 could be one of the mechanisms by which GCN2 promotes the induction of autophagy in response to leucine deprivation. However, short-term lysine deficiency or L-histidinol treatment induced autophagy but was not associated with decreased [S757]-ULK1 phosphorylation (nor with a decrease in [T389]-S6K1 phosphorylation). This observation suggests that GCN2 activation could affect ULK1 activity through a modification other than Ser757 dephosphorylation. Indeed, ULK1 undergoes multiple post-translational modifications (phosphorylations, acetylations, ubiquitylations) [34,65,66,^71–73^], which can affect its activity or stability. It is thus conceivable that one (or more) of these post-translational modifications could be controlled by GCN2 signaling. Furthermore, it was recently shown that in some cases of selective autophagy (activated specifically to degrade defective cellular components), the upregulation process involves an increase in ULK1 activity that does not appear to require dephosphorylation of its Ser757 residue [74–76]. We cannot rule out the possibility that the lack of a single EAA could be associated with the regulation of selective autophagy.

Our data reinforce the idea that the pathways involved in sensing AA availability can interact and are differently regulated depending on the nature of the stimulus (the missing EAA) [47,^77^]. Using mammalian cells, we observed that the withdrawal of all AA in the medium induced both GCN2 activation and mTORC1 inhibition together with a marked upregulation of autophagy which did not require GCN2 (Fig. S5). The induction of autophagy resulting from leucine deprivation required GCN2 activation and was associated with mTORC1 inhibition whereas autophagy induction in response to short-term lysine starvation was only associated with GCN2 activation. Thus, interactions and involvements of the different signaling pathways regulated by AA availability in the control of autophagy are complex. In fission yeast, Gcn2 has been shown to be required for upregulating autophagy in response to deprivations of leucine or all AA [78], notably through TORC1 inhibition [79]. Thus, the regulation of autophagy by GCN2 shares common features between yeast and mammalian cells. However, discrepancies can also be observed, likely related to differences in the dependence towards AA availability in the medium [80]. Further investigations will be required to precisely identify the molecular mechanisms linking GCN2 and TORC1 and determine their implications in the regulatory mechanisms of autophagy induction during AA deficiencies.

In conclusion, our data show that GCN2-eIF2α signaling is required to upregulate autophagy in response to short-term deprivations of a single EAA. This mechanism is associated with increased phosphorylation of [S278]-ATG16L1, reflecting ULK1 activity. In the rapid response to an EAA deficiency, this pathway could promote both autophagy induction and partial inhibition of protein synthesis, allowing the cell to manage its levels of the limiting EAA, and maintain basal protein synthesis for vital functions. Furthermore, our previous data indicated that the GCN2-eIF2α-ATF4 pathway upregulates the transcription of a number of autophagy genes [43], an effect that can be expected to help maintaining a high level of autophagy during stress. Taken together, our data thus indicate that GCN2 contributes to the adjustment of protein and AA homeostasis in response to stress induced by a deficiency in one or more EAA, a process that is essential for cell adaptation and survival.

## Materials and methods

### Reagents and media

All standard chemicals and the following were from Sigma-Aldrich: Dulbecco’s modified eagle’s medium (DMEM) (D6546), Dulbecco’s modified eagle’s medium/Nutrient F-12 Ham’s (DMEM/F12) (D9785), penicillin-streptomycin (P0781), L-glutamine (G8540), L-methionine (M5308), L-lysine (L8662), L-leucine (L8912), Protease Inhibitor Cocktail (PIC; P8340), Phosphatase Inhibitor Cocktail (PhIC; P0044), Normal goat serum (NGS; G9023), N,N-Bis-(2-hydroxyethyl)-2-aminoethanesulfonic acid, N,N-Bis-(2-hydroxyethyl)-taurine (BES; B9879), 3-Methyladenine (3-MA; M9281), lactacystin (Lacta; 426100), chloroquine (CQ; C6628). L-histidinol (Hol; H6647), Polybrene (TR-1003), 2-Mercaptoethanol (63689), puromycine (P9620), EGTA (E3889), CHAPS (C9426), sodium deoxycholate (D6750), Triton X-100 (T8787). Phosphate-buffered saline (PBS), Tris-buffered saline (TBS), Tris-glycine buffer (TG) and Tris-glycine Sodium Dodecyl Sulfate buffer were from Euromedex (ET330-A, ET220-B, EU0550 and EU0510, respectively). DMEM without amino acids was from Genaxxon (C4359). MEM non-essential amino acid solution (MEM-NEAA) and fetal bovine serum (FBS) were from Gibco-Fisher Scientific (10270.106 and 11140, respectively); before use in experiments of AA deprivation, FBS was dialyzed (3500-Da cut-off) against PBS (pH 7.4) at 4 °C.

### Antibodies

Anti-phospho-[T899]-GCN2 (ab75836), anti-phospho-[S51]-eIF2α (ab32157), anti-phospho-[S278]-ATG16L1 (ab195242) and anti-ATG16L1 (ab187671) antibodies were from Abcam. Antibodies targeting GCN2 (3302), eIF2α (9722), phospho-[Thr389]-S6K1 (9205), S6K (9202), phospho-[S757]-ULK1 (6888) and ATF4 (11815) were from Cell Signaling Technology. Anti-LC3B antibody used for western-blot analyses was from Novus (NB100-2220). For immunofluorescence, anti-LC3B (PM036) from MBL was used to stain LC3B. Anti-p62 antibody was purchased from Abnova (H00008878-M01). Anti-GFP and anti-rabbit Alexa-Fluor 488 (A11008) antibodies were from Invitrogen and anti-ULK1 (A7581) and anti-GAPDH (G9545) antibodies from Sigma-Aldrich. Anti-species secondary antibodies coupled to horseradish peroxidase (HRP) were from Cell Signaling Technology (anti-rabbit antibody 7074 and anti-mouse antibody 7076).

### Cell culture and treatments

Mouse embryonic fibroblasts (MEFs) deficient in GCN2 or ATF4 expression [37] and eIF2α ^S51A/S51A^ MEFs [81] were kindly provided by Dr. D. Ron and Dr. H. Harding (Institute of Metabolic Science, Cambridge, UK) and Dr. R. J. Kaufman (University of Michigan, La Jolla, California, USA), respectively. All cell cultures were maintained at 37 °C in the presence of 5% CO2 (v/v) in DMEM containing 10% FBS, 2 mM L-glutamine, NEAA, 100 units/ml penicillin and streptomycin. As recommended, all culture media for maintenance and treatments of MEFs were supplemented with 50 µM of 2-Mercaptoethanol as an antioxidant. All MEFs were at a passage less than 12 in all experiments, with no more than two passages of difference between MEFs of different genotypes. For all experiments, cells were plated at desired confluence 48 h before treatment. At 1 h before treatment, cells were replenished with fresh medium, as previously described by Tian et al. [52]. For experiments of deprivation of a single EAA, a DMEM/F12 medium devoid of leucine, glutamine, lysine and methionine (4-AA-devoid DMEM/F12) was used to make the control (Ctr) medium and media lacking L-leucine (-Leu) or L-lysine (-Lys). The commercial medium was therefore supplemented with L-glutamine, L-methionine and either L-lysine (for medium lacking L-leucine) or L-leucine (for medium lacking L-lysine) in the presence of 10% (v/v) dialyzed FBS. Cells were washed once with pre-warmed PBS and incubated in pre-warmed Ctr or -Leu or -Lys medium for the indicated time. For L-histidinol (Hol) treatment, a concentrated solution (50 mM) was extemporaneously prepared in PBS and diluted in the medium (4 mM final). The pH was adjusted to the same value as that of the Ctr medium. All cell culture experiments were repeated at least three times.

### Generation of cell lines

*Gcn2* KO MEFs stably expressing GCN2 or GFP as a control were generated by retroviral infection. Retroviral vector encoding C-terminally FLAG tagged mouse *Gcn2* was obtained from addgene (pMXs-m*Gcn2*-FLAG-IP, Plasmid 101794; Depositor: Dr. Oyadomari Lab) and has been previously used in another study [82]. Plat-E producer cells were used to generate retroviral stocks. Transfection for retrovirus production was performed using FuGENE® 6 Transfection Reagent (Promega, E2691). Retroviral transduction was performed on *gcn2* KO MEFs 24 h after in presence of 5 ug/mL of Polybrene for 6 hours. Two days later, puromycin treatment started for selection during 6 days.

### RNA interference

MEFs were transfected using a CaCl_2_-BES method. *Atf4* siRNA (*Atf4* target sequence 5’-CTGGAGTTAGTTTGACAGCTA-3’) or scrambled sequence was brought into the reaction mixture 10 min before application to the cells at a final concentration of 20 nM. After overnight incubation, the cells were rinsed and leucine or lysine starvation started 2 to 6 h after. *Atf4* siRNA and scrambled sequence (1027281) were from Qiagen.

### Mice, nutritional protocol, and treatments

The mice used in this study were from the C57BL/6J strain. They were housed in an animal facility at INRAE with a 12-h light/dark cycle at 22 °C. *gcn2* knock-out (KO) mice were kindly provided by Prof. D. Ron (Institute of Metabolic Science, Cambridge, UK). The production of *gcn2* KO mice was previously described [2]. *gcn2* KO male animals with *gcn2* ^+/+^ and *gcn2* ^−/−^ genotypes were derived by crossing +/- animals. Animals had *ad libitum* access to food and water at all times, unless otherwise indicated.

Experimental diets were manufactured in our own facility (Experimental Food Preparation Unit, INRAE), and nutritional experiments were performed as previously described [2]. Briefly, the nutritional deficiency in an EAA is carried out by means of experimental diets containing no proteins but 20% of free AA. The mice were previously fed with the control experimental diet for one week. On the evening before the day of the experiment food was withdrawn for the duration of the night (15 h), so that the next morning mice would significantly consume the experimental diet lacking one EAA (leucine-devoid diet). This protocol allows a significant decrease in the blood concentration of the EAA missing from the diet, as previously described [2]. This decrease is a prerequisite for GCN2 activation. Animals were sacrificed and livers removed, snap frozen and stored at -80 °C.

### Plasma amino acid analysis

Cardiac blood samples were drawn from anaesthetized mice, and plasma were prepared. Plasma samples were treated with sulfosalicylic acid and thiodiglycol. Free-AA proportions were determined using ion-exchange liquid chromatography followed by post-column detection with ninhydrin (L-8900 Amino Acid Analyzer, Hitachi High-Technologies Corporation, Tokyo, Japan). The internal standard, norleucine, was used to measure sample treatment efficiency, and correct the crude values.

### Protein degradation measurement

Protein degradation was measured by radiolabeling cellular proteins with L-[^35^S]Met and using a pulse-chase approach, as previously described [49]. Briefly, cells were incubated for 16 h in a labeling medium containing 7 µCi of L-[^35^S]Met/ml to label cellular proteins. Then, cells were rinsed with culture medium to remove the labeled compound before adding excess of unlabeled precursor (L-Met) for successive chases. Cells were incubated for 2 x 1h in fresh complete medium, before a further 2-h period in control or leucine-free medium. Proteolysis rate was calculated from radioactivity measurements in the medium (resulting from released extracellular L-[^35^S]Met). Results are expressed as variation in proteolysis rate in response to leucine deficiency compared to control proteolysis rate (control medium).

### Electron microscopy

Samples were fixed with 2.5% glutaraldehyde and then in 1% OsO_4_ (diluted in phosphate buffer) prior to dehydration in alcohol and propylene oxide. After overnight impregnation with a mixture of propylene oxide and Epon, samples were soaked in a resin that was included in capsules and allowed to polymerize. Then, the resin blocks were then cut into ultra-fine sections for observation under a JEOL 1010 electron microscope (JEOL USA).

### Protein extraction and western-blot analysis

Protein were extracted from cultured cells and liver samples with 1X Laemmli buffer, as previously described by Tian et al. [52]. Alternatively, another extraction buffer has also been used in some experiments (150 mM NaCl, 50 mM Tris-HCl pH 7.4, 2.5 mM EGTA, 1% Triton X-100, 0.15% CHAPS, 0.5% sodium deoxycholate, 1 mM Dithiothreitol [DTT {Invitrogen, 15508013}], 1X PIC and 1X PhIC). Samples were boiled at 95 °C for 5 min and then resolved by SDS-PAGE on 8%, 10% or 14% polyacrylamide gels in TG-SDS buffer. Immunoblot conditions for [S278]-phospho-ATG16L1 detection were based on those described by Tian et al. [52]. After protein transfer in TG buffer onto polyvinyl-dimethylsulfoxide (PVDF) membranes (Amersham Hybond P0.45, 10600023), the membrane portions intended for [S278]-phospho-ATG16L1 detection were saturated with 2% (w/v) bovine serum albumin (BSA; Fischer Scientific BP9702-100) in TBS containing 0.1% Tween-20 (Sigma-Aldrich, P7949) (TBS-T), for 30 min at room temperature. All other membranes were blocked with non-fat dried milk powder (5% w/v) diluted in TBS-T for 1 h. Primary antibodies were diluted according to the manufacturer’s instructions in TBS-T containing 2% (anti-[S278]-phospho-ATG16L1) or 5% BSA (all other antibodies). Membranes were incubated with primary antibodies during 2 h at room temperature or overnight at 4 °C, then washed 3 times for 5 min in TBS-T. HRP-coupled anti-species secondary antibodies were diluted at 1:2000 in TBS-T containing 5% (w/v) non-fat dried milk and used at room temperature for 1 h. Membranes were washed 2 times for 5 min in TBS-T, then rinsed in TBS before analysis. Luminata western HRP substrate (Millipore, WBLUR0500) and a chemiluminescence imager (G:box, Syngene) were used to detect signals. GAPDH expression levels served as internal standard for protein loading. Signal intensities were quantified using the Image J software. For a given sample, the respective intensities of LC3-I and LC3-II signals were always quantified from the same selected area. Relative quantifications have been performed, with control condition values normalized to 1 so as to be able to compare the extent of treatment effects in between all experiments.

### LC3 immunofluorescence

The cells were seeded in eight-well culture chambers (Falcon, 354108). At 1 h before treatment, cells were replenished with fresh medium, then placed either in control or leucine-devoid medium for 2 h. This experiment was performed in presence of chloroquine (CQ, 20 µM) that inhibits the fusion of the autophagosome to the lysosome, thereby permitting the quantitation of the autophagosome formation as a measure of the degree of autophagy by arresting autophagic flux before the lysosomal degradation can occur [83]. We have previously shown that supplementation with chloroquine does not impede the activation of autophagy upon leucine starvation [43]. After treatment, at room temperature, cells were rinsed twice with PBS, fixed in 4% paraformaldehyde in PBS for 10 min and permeabilized by incubation for 45 min in PBS containing 0.5% Triton X-100 and 5% NGS (to saturate aspecific antigenic sites). Cells were incubated with anti-LC3 antibody (1:200 in PBS containing 0.5% Triton X-100 and 0.5% NGS) for 1 h at room temperature followed by overnight at 4 °C. After PBS washing, the primary antibody was hybridized with an anti-species secondary antibody coupled to Alexa-Fluor 488. After washing three times with PBS, cells were mounted with Vectashield Antifade Mounting Medium (Vector Laboratories, H-1000-10) and viewed under a Zeiss Fluorescence microscope. Quantification of punctae per cell was performed on 40-50 cells per treatment condition in three independent experiments using Image J software.

### Statistical analyses

Data are presented as mean ± standard error of the mean (SEM). Non-paired Student’s t-tests were used to statistically compare data between treated- and control groups for a given cell type. Differences were considered significant with p <0.05.

### Ethics statement

Animal experiments were carried out in accordance with INRAE guidelines in compliance with European animal welfare regulation. Mice maintenance and all experiments have been approved by our institutional animal care and use committee, in conformance with French and European Union laws (permission to experiment on mice APAFIS#5558-2016060314257755 v5, local ethic committee C2EA-02, animal facilities agreement D6334515, GMO agreement #4713).

### Health and safety statement

All mandatory laboratory health and safety procedures have been complied with in the course of conducting any experimental work reported in this paper.

## Abbreviations

AA: amino acid; ATF4: cyclic AMP-dependent transcription factor ATF-4; *Atg*: Autophagy-related gene; ATG16L1: autophagy-related protein 16- 1; Ctr: control; EAA: essential amino acid; eIF2α: eukaryotic translation initiation factor 2 subunit alpha; GAPDH: glyceraldehyde-3-phosphate dehydrogenase; GCN2: eIF-2-alpha kinase GCN2; GFP: green fluorescent protein; Hol: L-histidinol; KO: knock-out; Leu: leucine; Lys: lysine; MAP1LC3B/LC3B: microtubule-associated protein 1 light chain 3 beta; MEFs: mouse embryonic fibroblasts; mTORC1: serine/threonine-protein kinase mTOR complex 1; p62/SQSTM1 (sequestosome-1); S6K1: ribosomal protein S6 kinase beta-1; ULK1: serine/threonine-protein kinase ULK1; WT: wild-type.

## Supplementary Material

Supplemental Material

## Data Availability

The data that support the findings of this study are included within the article or its supplementary materials.
