# Supplementary material for: GCN2 upregulates autophagy in response to short-term deprivation of a single essential amino acid
Source: Autophagy Rep. 2022 Apr 7;1(1):119–42. doi: 10.1080/27694127.2022.2049045 (PMC11864613; doi:10.1080/27694127.2022.2049045)
Supplement: Supplemental Material [file KAUO_A_2049045_SM4089.docx]

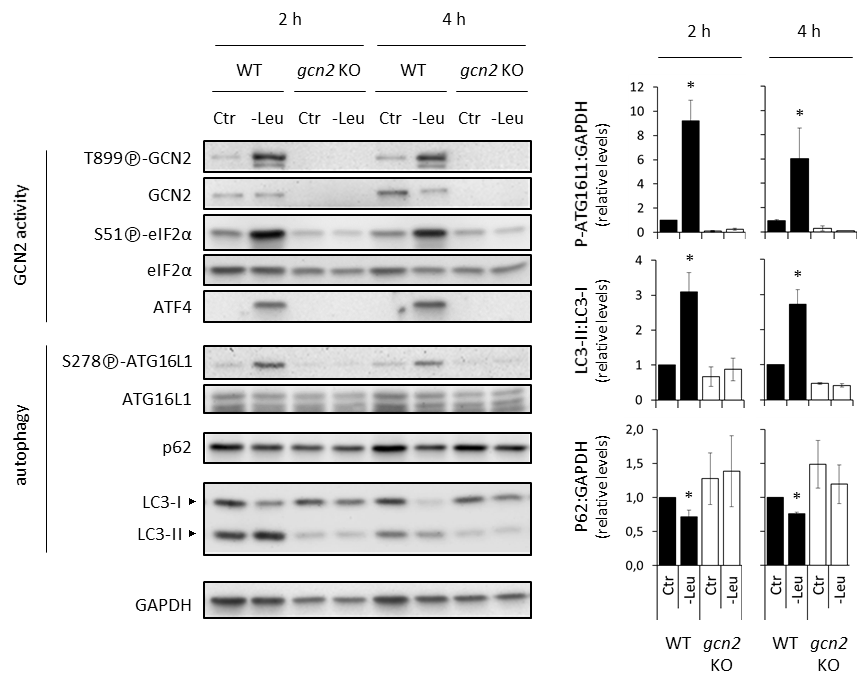


**Figure S1.** Time-course analysis of GCN2 activity and autophagy markers in response to leucine deprivation in MEFs. WT and *gcn2* KO MEFs were maintained in Ctr or -Leu medium for 2 or 4 h and total protein extracts were analyzed by immunoblotting. Representative immunoblots and relative quantifications of P-[S278]-ATG16L1 to GAPDH, LC3-II to LC3-I and p62 to GAPDH are shown (three independent experiments). Bar values are mean ± SEM (*, p < 0.05 relative to Ctr of the same cell type, Student’s t-test).


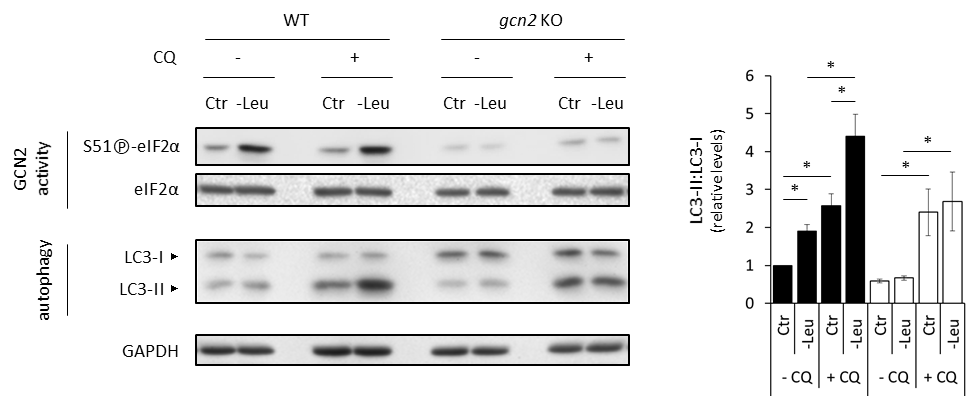


**Figure S2.** The increase in autophagic flux resulting from short-term leucine deprivation was compromised in *gcn2* KO cells. Immunoblot analyses of total protein extracts of WT and *gcn2* KO MEFs either kept in Ctr or -Leu medium in the absence or presence of chloroquine (CQ, 20 µM) for 1 h. Immunoblots of one representative experiment are shown. Relative quantification of LC3-II to LC3-I was performed from results of four independent experiments. Bar values are mean ± SEM (*, p < 0.05 relative to controls of the same cell type, Student’s t-test).


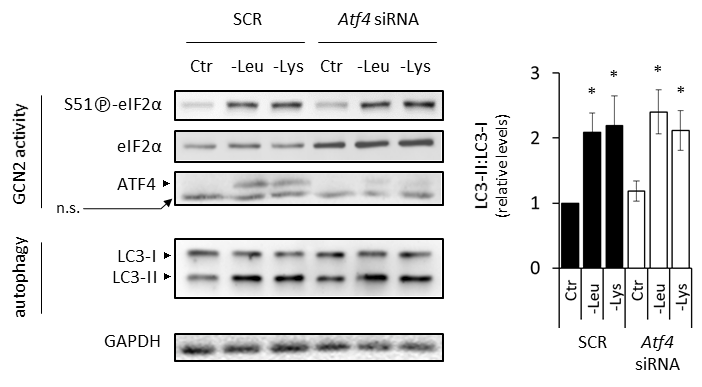


**Figure S3.** Down-regulation of ATF4 expression did not impede LC3 conversion in response to 2 h leucine or lysine deprivation in MEFs. Scrambled sequence (SCR) or *Atf4* siRNA-treated WT MEFs were cultured for 2 h in Ctr or -Leu or -Lys medium and total protein extracts were analyzed by immunoblotting. Representative immunoblots and relative quantification of LC3-II to LC3-I are given (three independent experiments). Bar values are mean ± SEM (*, p < 0.05 relative to Ctr with the same RNA sequence, Student’s t-test).


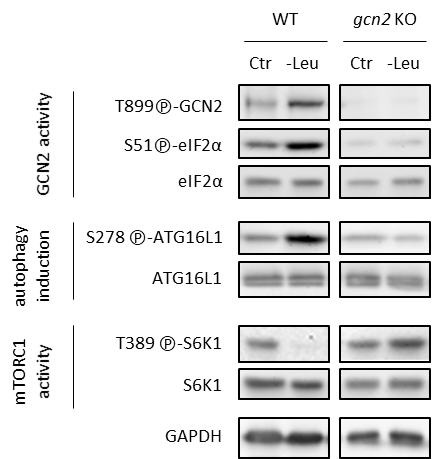


**Figure S4.** The GCN2-dependent phosphorylation of ATG16L1 resulting from short-term leucine-deprivation was associated with GCN2-dependent inhibition of mTORC1 activity. WT and *gcn2* KO MEFs were either kept in Ctr or -Leu medium for 1 h and total protein extracts were analyzed by immunoblotting. Representative data are shown (three independent experiments).


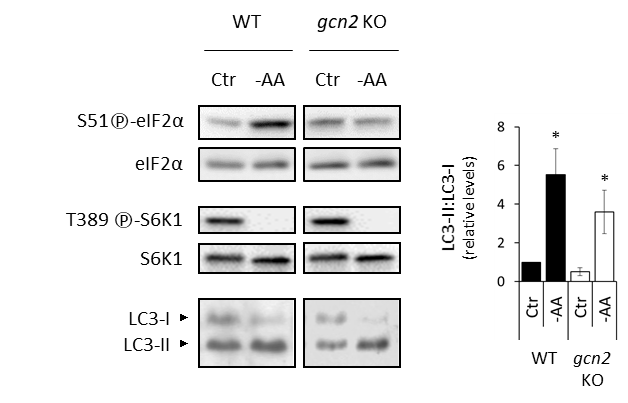


**Figure S5.** GCN2 was not required for upregulating autophagy in response to 1 h withdrawal of all AAs in MEFs. Immunoblot analyses of total protein extracts of WT and *gcn2* KO MEFs either kept in Ctr or -AA medium for 1 h. Representative immunoblots and relative quantification of LC3-II to LC3-I are given (three independent experiments). Bar values are mean ± SEM (*, p < 0.05 relative to Ctr of the same cell type, Student’s t-test).
